# Structure and Mechanical Properties of As-Cast Ti–5Sn–*x*Mo Alloys

**DOI:** 10.3390/ma10050458

**Published:** 2017-04-27

**Authors:** Hsing-Ning Yu, Hsueh-Chuan Hsu, Shih-Ching Wu, Shih-Kuang Hsu, Wen-Fu Ho

**Affiliations:** 1 Department of Surgery, Division of Orthopaedics, Zuoying Branch of Kaohsiung Armed Forces General Hospital, Kaohsiung 81342, Taiwan; david4229@mail.ngh.com.tw; 2 Department of Dental Technology and Materials Science, Central Taiwan University of Science and Technology, Taichung 40601, Taiwan; hchsu@ctust.edu.tw (H.-C.H.); scwu@ctust.edu.tw (S.-C.W.); sksheu@ctust.edu.tw (S.-K.H.); 3 Department of Chemical and Materials Engineering, National University of Kaohsiung, Kaohsiung 81148, Taiwan

**Keywords:** titanium alloys, microstructure, mechanical properties

## Abstract

Ti–5Sn–*x*Mo (*x* = 0, 1, 3, 5, 7.5, 10, 12.5, 15, 17.5, and 20 wt %) alloys were designed and prepared for application as implant materials with superior mechanical properties. The results demonstrated that the crystal structure and mechanical properties of Ti–5Sn–*x*Mo alloys are highly affected by their Mo content. The as-cast microstructures of Ti–5Sn–*x*Mo alloys transformed in the sequence of phases α′ → α″ → β, and the morphologies of the alloys changed from a lath structure to an equiaxed structure as the Mo content increased. The α″-phase Ti–5Sn–7.5Mo (80 GPa) and β-phase Ti–5Sn–10Mo (85 GPa) exhibited relatively low elastic moduli and had excellent elastic recovery angles of 27.4° and 37.8°, respectively. Furthermore, they exhibited high ductility and moderate strength, as evaluated using the three-point bending test. Search for a more suitable implant material by this study, Ti–5Sn–*x*Mo alloys with 7.5 and 10 wt % Mo appear to be promising candidates because they demonstrate the optimal combined properties of microhardness, ductility, elastic modulus, and elastic recovery capability.

## 1. Introduction

Titanium (Ti) and its alloys have been widely applied in orthopedic and dental implants due to their high biocompatibility, superior corrosion resistance, and adequate mechanical properties [1]. Commercially pure titanium (c.p. Ti) with lower strength is currently used in dentistry, and Ti–6Al–4V ELI alloy with relatively high strength is used in high stress-bearing situations. Ti–Ni alloys exhibiting unique shape memory effect and superelasticity are suitable for biomedical applications such as orthodontic arch wires, bone plates, and vascular stents [2]. However, questions have been raised about the cytotoxic and even carcinogenic risks that these biometals pose to the human body because of the release of Al, V, and Ni [3,4,5]. Over the past few years, numerous new Ti alloys with improved mechanical properties have been developed by alloying Ti with nontoxic elements, such as Ti–Nb–Ta–Zr [6], Ti–Zr–Sn–Mo–Nb [7], Ti–Nb–Mo [8], and Ti–Nb–Fe [9].

However, another problem must be overcome regarding conventional commercial biometals: the stiffness mismatch between bone and Ti implants. This biomechanical incompatibility can lead to a stress shielding effect, eventually contributing to detrimental bone resorption and artificial implant failure [10,11]. Therefore, a reduction of the elastic modulus is a major goal of new Ti alloys designed for surgical implant applications. One method to alleviate the problem is to optimize the mechanical properties through microstructure and phase control in Ti alloys by adding nontoxic and nonallergic β stabilizer elements. Niinomi [12] suggested that β-type Ti alloys are much less stiff than α- or α+β-type alloys. The β-type Ti alloys, therefore, have received considerable attention as biometals. Additionally, Ho et al. demonstrated that the α″ phase exhibited a significantly lower modulus in Ti–Mo alloy systems [4]. Similar results were obtained with other ternary Ti alloys [8,13,14].

Sn is a biocompatible element; the addition of Sn can enhance cold workability of Ti alloys [15]. In a Ti–Sn binary alloy system with 1 to 30 wt % Sn, all the binary Ti alloys exhibited the α structure, and their elastic moduli decreased with increasing the Sn concentration [16]. Furthermore, the addition of Sn can produce an apparent improvement in the grindability of Ti alloys, and the alloys with greater Sn contents could be ground more easily [17]. Similar results were also obtained on the machinability of a Ti–Sn binary system [18]. Nonetheless, much effort is still needed to enhance mechanical properties for biomedical implant applications such as a lower elastic modulus and higher strength. Mo is a superior choice as a β stabilizer alloying element that can be effective in reducing the elastic moduli of Ti alloys [19,20]. Mo is also a nontoxic and hypoallergenic element [19,21]. Previous research also demonstrated that Ti–Mo alloys possess superior corrosion resistance and biocompatibility [22,23]. The primary goal of this study is to investigate the effects of Mo on the microstructure and mechanical properties of a Ti–5Sn-based alloy for potential biomedical and dental implant applications.

## 2. Materials and Methods

Experimental Ti–5Sn–*x*Mo (*x* = 1, 3, 5, 7.5, 10, 12.5, 15, 17.5, and 20) (in wt %) alloys were fabricated from grade 2 Ti (99.7% in purity), Sn (99.95% in purity), and Mo (99.95% in purity) by using arc melting and a vacuum pressurized casting system under argon atmosphere. The ingots were flipped after each melting step and remelted five times before casting to obtain chemical homogeneity. The mean diameter and height of the button-like alloy ingot (13 g) was approximately 22 and 9 mm, respectively. Each metal ingot was melted again in a copper crucible before casting, and then the molten alloy was quickly poured into a room temperature graphite mold. The specimen was cooled in a dry argon atmosphere for about 60 s in the casting chamber. A detailed description of the procedure can be found in our previous work [24].

Specimens were first metallurgically ground using standard techniques and then mechanically polished with 0.3 μm alumina powder. The specimens were then etched in a solution containing 80 vol.% H_2_O, 15 vol.% HNO_3_, and 5 vol.% HF. The prepared surfaces were then observed using an optical microscope (OM; BH2, Olympus, Tokyo, Japan) for microstructure observation and an X-ray diffractometer (XRD; XRD-6000, Shimadzu, Kyoto, Japan) for phase analysis. The X-ray diffraction patterns were performed by using Ni-filtered CuKα radiation operating at 30 kV and 30 mA at room temperature. The crystalline phases were identified by matching their characteristic peaks with the Powder Diffraction Standards (JCPDS) database.

Microhardness values of all the alloys were taken by applying a 100 g load and a 15 s dwell time using a microhardness tester (MVK-E3, Mitutoyo, Tokyo, Japan). Three specimens were used to test the microhardness of each alloy, and five tests were performed in randomly chosen positions for each specimen. In this study, the microhardness was not expressed in terms of the position. For dental or orthopedic devices such as dental implants and bone plate, flexural stress was loaded frequently during mastication and body weight loading, rather than tensile loading; therefore, in this work the bending test was adopted to assess the mechanical properties of all the test samples. A desktop mechanical tester (AG-IS, Shimadzu, Kyoto, Japan) was used to conduct a three-point bending test at a crosshead speed of 0.5 mm/min at room temperature, according to ASTM E855. Prior to the test, the surface of each test specimen was ground with SiC abrasive paper, producing a final specimen size of approximately 40 × 5.0 × 0.9 mm. The bending strength and modulus were determined according to the following equations [25]

(1) $$\sigma =3PL/2bh^{2},$$

where σ is the bending strength (MPa), *P* the load (N), *L* the span length (30 mm), *b* the specimen width (5.0 mm), and *h* the specimen thickness (0.9 mm).

(2) $$E=L^{3}\Delta P/4bh^{3}\Delta \delta ,$$

where *E* is the elastic modulus in bending (GPa), Δ*P* the load increment as measured from the preload (N), and Δδ the deflection increment at midspan, as measured from the preload. The elastic recovery angle for each alloy was examined from the change in deflection angle before and after unloading at the preset bending deflection of 8 mm. Experimental details can be found in Hsu et al. [26].

## 3. Results and Discussion

### 3.1. Phase Identification

**Figure 1:**
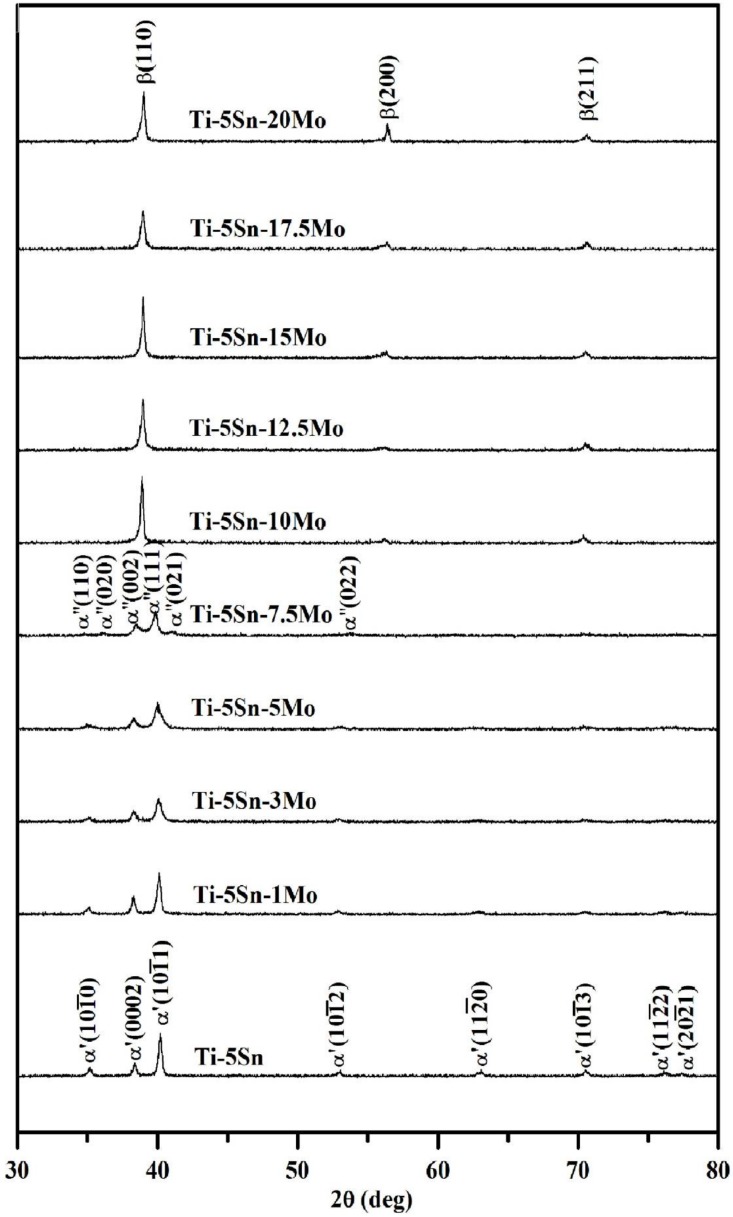
XRD patterns of as-cast Ti–5Sn and Ti–5Sn–*x*Mo alloys.

The phase structures of each as-cast alloy were analyzed using X-ray diffraction at room temperature. As illustrated in Figure 1, Ti–5Sn exhibits a hexagonal α′ phase, which was confirmed by our previous study [27]. The crystal structures of Ti–5Sn–*x*Mo alloys are shown to be highly dependent on their Mo content. With a 1 to 5 wt % Mo addition, the ternary alloys still consisted mainly of a single α′-Ti phase, while the orthorhombic α″-Ti peaks appeared completely in Ti–5Sn–7.5Mo alloy. Some other Ti alloy systems can also present the α″ phase, which can be formed directly from quenching without the aid of external stress [13,14]. An increase of Mo content to 10 wt % or greater, a bcc β phase was entirely retained owing to the β stabilizing effect of alloying element Mo [4,20]. A similar result was found as in an earlier work [8], wherein the β phase could be fully retained upon quenching at 10 wt % Mo in Ti–5Nb–*x*Mo alloy. The XRD peaks of the β-Ti alloys shifted toward the high angle side with increasing Mo content resulted from the differences in atomic radius between Ti and Mo. Because the atomic radius of Mo (1.40 Å) is smaller than that of Ti (1.47 Å) [28], the β phase lattice parameters decreased with the addition of Mo. The shift was more obvious for the higher Mo content, as indicated by the XRD data (Figure 1).

A study on binary Ti–Mo alloys indicated that athermal ω precipitations could be observed in the XRD profiles with increasing Mo content up to 9 wt % [20]. Among the ternary Ti–5Sn–*x*Mo alloys, there were no detectable ω peaks in the XRD patterns. It was found that alloying with an appropriate amount of Sn could effectively suppress the athermal ω phase precipitation in Ti alloys [29], such as Ti–Nb–Zr–Sn [30] and Ti–Nb–Sn [26] systems. In the present study, a 5 wt % Sn addition effectively suppressed the ω phase formation in the Ti–Sn–Mo system. The ω phase is likely to raise the elastic modulus and to bring about embrittlement of a Ti alloy [8,9,31]. Thus, its precipitation must generally be avoided.

### 3.2. Microstructure

**Figure 2:**
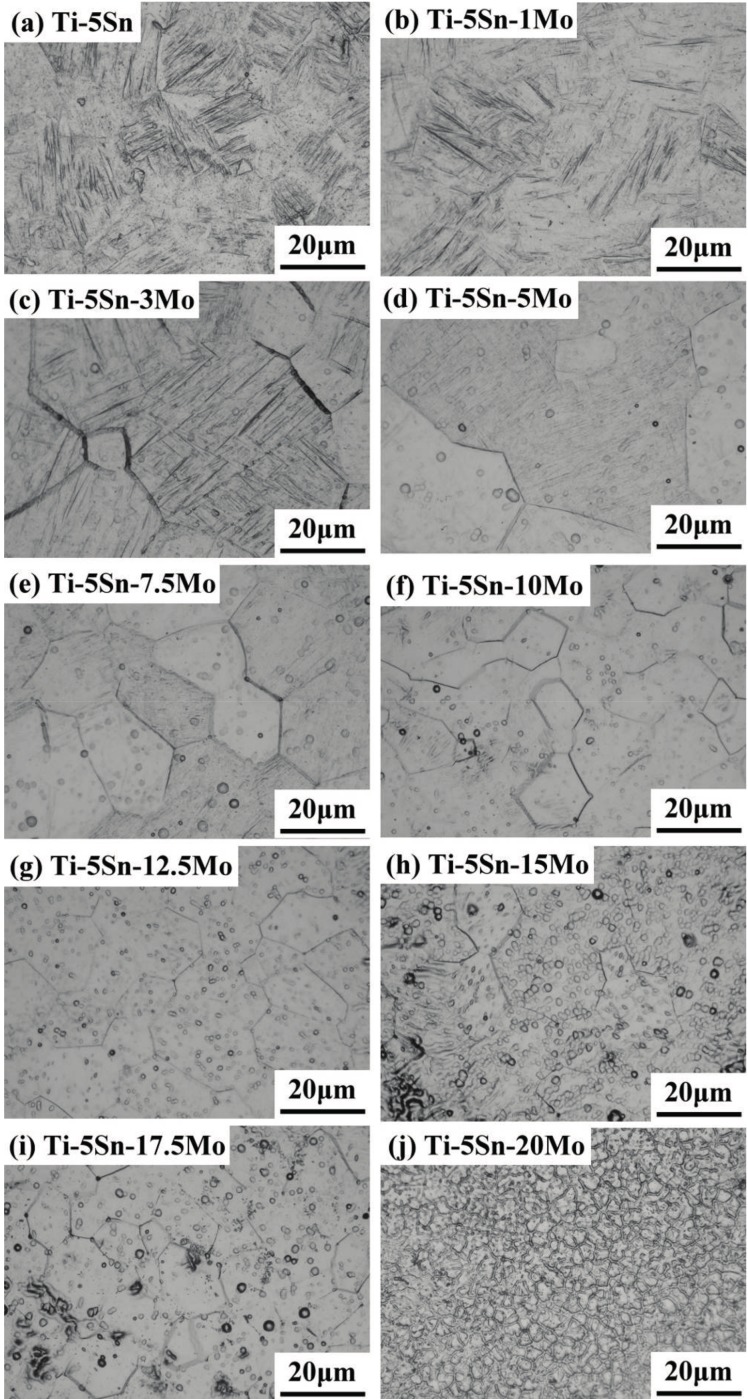
Light micrographs of as-cast Ti–5Sn and Ti–5Sn–*x*Mo alloys.

Figure 2 displayed the optical microstructures of as-cast Ti–5Sn and Ti–5Sn–*x*Mo alloys. The Ti–5Sn alloy showed coarse lath-like α′ phase precipitates. Figure 2b–d indicated that the α′ phase was observed in Ti–5Sn–*x*Mo alloys with 1, 3, and 5 wt %. These alloys also exhibited a lath structure; finer laths appeared with the increased content of Mo. When the Mo content was 7.5 wt %, a relatively acicular-like α″ phase structure was examined, as illustrated in Figure 2e. When 10 wt % or greater Mo was added, an equiaxed retained β phase became the dominant phase (Figure 2f–j). Moreover, the average grain size of the β phase decreased as the alloying Mo content increased. This was possibly caused by solute–grain boundary interactions that retarded the grain growth. At the 20 wt % Mo composition point, the casting dendritic substructure generated by the solidification process was visible in the β grains, as displayed in Figure 2j.

### 3.3. Mechanical Properties

**Figure 3:**
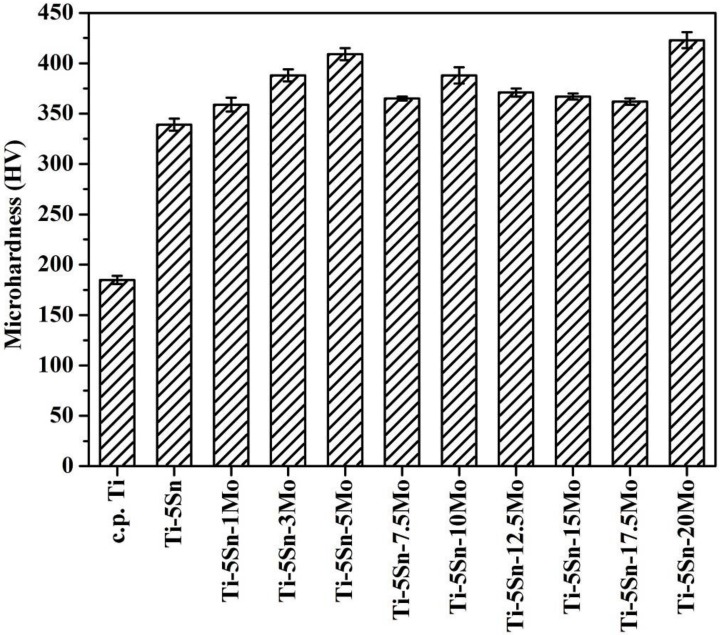
Microhardness of as-cast c.p. Ti, Ti–5Sn, and Ti–5Sn–*x*Mo alloys.

Microhardness values of as-cast c.p. Ti, Ti–5Sn, and Ti–5Sn–*x*Mo alloys were tested and presented in Figure 3. C.p. Ti has a significantly lower microhardness (186 HV) that is obviously lower than those of all the Ti–5Sn–based alloys (339–423 HV). The Mo element can improve the microhardness in Ti–5Sn-based alloys because of the solid solution effect, crystal structure or phase (α′, α″, β). Hence, the ternary Ti–5Sn–*x*Mo alloys (360–423 HV) exhibited greater microhardness than the binary Ti–5Sn alloy (339 HV). By adding 1, 3, or 5 wt % Mo to Ti–5Sn, the hardness increased substantially to 360, 389, and 409 HV, respectively. The Ti–5Sn–7.5Mo with the α″ phase had slightly lower hardness, which is because α″ phase involves smaller strains than those required to form dislocated α′ phase during phase transition. Similar results have been found in other Ti alloys, such as Ti–Mo [4], Ti–Nb [32], and Ti–Mo–Cr [14]. Furthermore, Ti–5Sn–20Mo (423 HV) had the greatest microhardness in the present study, which was greater than those of the Co–Cr alloy (350–390 HV) [33] and Ti–6Al–4V alloy (380 HV) [34].

**Figure 4:**
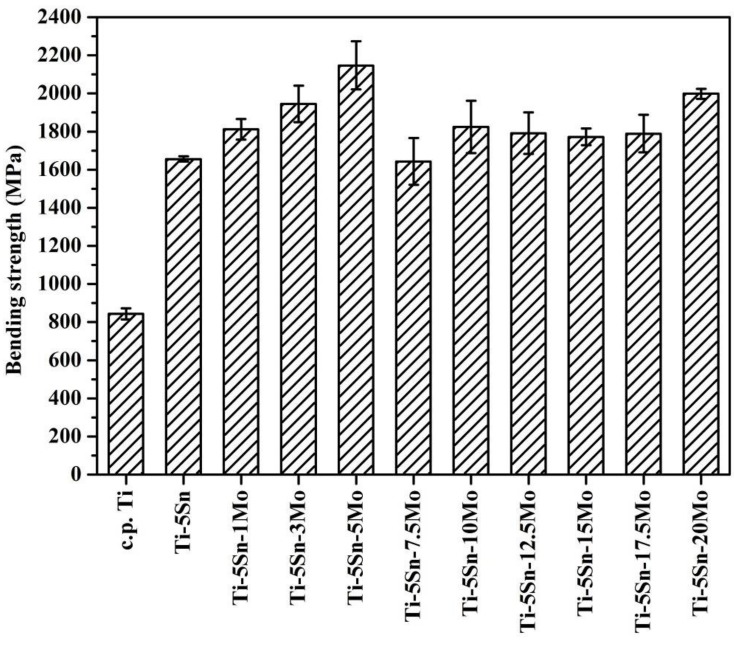
Bending strengths of as-cast c.p. Ti, Ti–5Sn, and Ti–5Sn–*x*Mo alloys.

The bending strength of as-cast c.p. Ti and Ti–5Sn-based alloys are plotted in Figure 4. As expected, the variation in strengths of these alloys is similar to the trend of the microhardness. The bending strengths of all the Ti–5Sn-based alloys were significantly greater (1643–2147 MPa) than that of c.p. Ti (844 MPa). The strengths of Ti–5Sn–1Mo, Ti–5Sn–3Mo, and Ti–5Sn–5Mo alloys gradually increased with an increase in the Mo content caused by a stronger solution strengthening effect, though they have the same crystal structure (α′ phase) as Ti–5Sn. Note that Ti–5Sn–7.5Mo alloy had a relatively low strength, which is considered a result of the smaller strains of the α″ martensitic structure. Additionally, Ti–5Sn–20Mo exhibited the greatest strength among the Ti–5Sn-based alloys with β phase, which is partially a result of the higher Mo content. Furthermore, the Hall–Petch relation can account for the strength of the Ti–5Sn–20Mo alloy with decreasing grain size, resulting in increased strength of the alloy.

**Figure 5:**
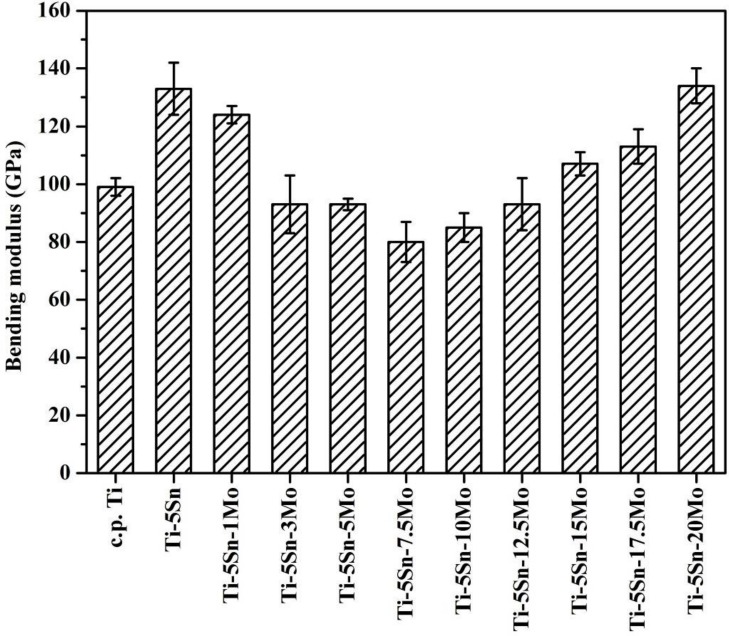
Bending moduli of as-cast c.p. Ti, Ti–5Sn, and Ti–5Sn–*x*Mo alloys.

Figure 5 illustrates the composition dependence of the bending elastic modulus for the as-cast c.p. Ti and Ti–5Sn-based alloys. The results indicated that the tendency of the bending modulus with Mo content was not in accordance with that of the bending strength or microhardness. The elastic modulus is an intrinsic property of materials and is particularly sensitive to phases and crystal structures than are other factors. As displayed in Figure 5, Ti–5Sn had a high bending modulus (133 GPa) among the metals tested in the present study. When 1, 3, or 5 wt % Mo was added, the modulus of alloys with the same α′ structure gradually decreased from 124 to 93 GPa. Ti–Nb system also exhibited a similar behavior. Here it is worth noting that the Ti–5Sn–7.5Mo alloy with α″ martensitic structure exhibited the lowest modulus. It was proven that the α″ phase contributed to the lower elastic modulus in many Ti alloy systems, including Ti–Mo [4], Ti–Nb–Mo [8], Ti–Zr–Mo [13], and Ti–Mo–Cr [14] alloys. Among the Ti–5Sn–*x*Mo alloys with a β phase, the Ti–5Sn–10Mo (85 GPa) had the lowest modulus, while the Ti–5Sn–20Mo (134 GPa) had the highest. This result demonstrates that the metastable β phase possesses a much lower elastic modulus than a highly stable one with higher β stabilizer content. This may conclude that the phase stability must be one of the major factors to determine the elastic modulus of a Ti alloy [35].

**Figure 6:**
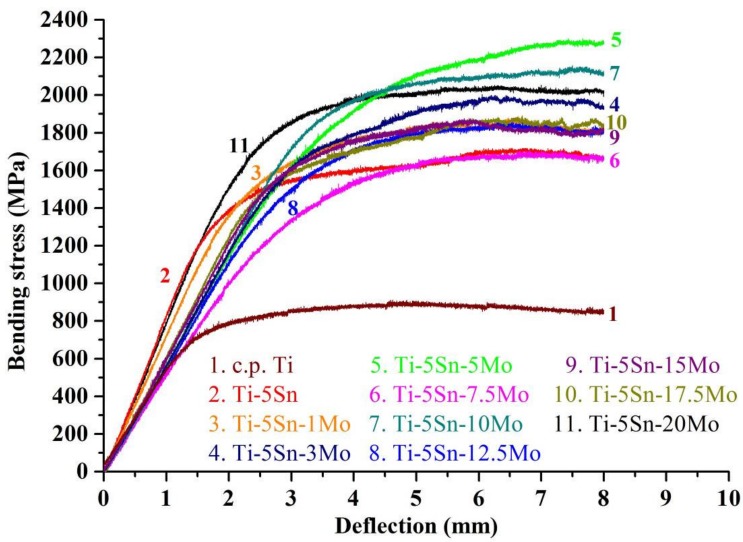
Bending stress-deflection profiles of as-cast c.p. Ti, Ti–5Sn, and Ti–5Sn–*x*Mo alloys.

The typical bending stress-deflection profiles of c.p. Ti and Ti–5Sn-based alloys are displayed in Figure 6. Fracturing was not observed on all samples after being loaded up to the preset maximum deflection of 8 mm, exhibiting high ductility. The mechanical performance of Ti alloys intended to be used in orthopedic and dental implants is generally examined by the ratios of bending strength to modulus (×1000) [8]. Potential candidates for implant metals should have much higher strength-to-weight ratios [36]. In the current study, the α′-phase Ti–5Sn–5Mo alloy showed the highest ratio of strength to modulus (23.1); it was substantially greater than that of c.p. Ti (8.5) and of the Ti–5Sn alloy (12.5). A relatively low modulus coupled with a high strength is an important property of Ti alloy for stress-bearing orthopedic applications; however, a low elastic modulus is a more crucial target to avoid stress shielding. In comparison with Ti–5Sn–5Mo, the α″-phase Ti–5Sn–7.5Mo alloy with the lowest bending modulus was anticipated to be used in heavy load-bearing implants, although its strength-to-modulus ratio (20.5) was slightly lower because of its lower strength. Hence, the development of a new Ti–5Sn–7.5Mo alloy with improved properties, and strength in particular, should be the next step for this research.

**Figure 7:**
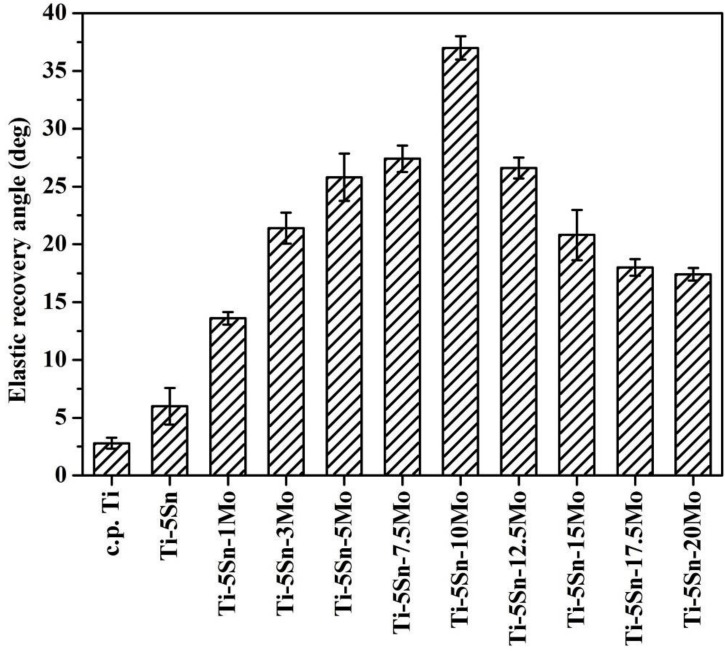
Elastic recovery angles of as-cast c.p. Ti, Ti–5Sn, and Ti–5Sn–*x*Mo alloys.

As illustrated in Figure 7, the Ti–5Sn–7.5Mo alloy exhibited a greater elastic recovery angle (27.4°), which was much greater than that of c.p. Ti (2.7°) and of Ti–5Sn (6.0°). It is noteworthy that Ti–5Sn–10Mo (37.8°) exhibited a significantly greater elastic recovery angle because of its high strength-to-modulus ratio (21.5). Accordingly, the β-phase Ti–5Sn–10Mo appears to be another promising candidate for biomedical implants due to its low modulus, superior elastic recovery capability, and appropriate strength.

## 4. Conclusions

The present study was mainly to evaluate the structure and mechanical properties of as-cast Ti–5Sn–*x*Mo alloys with various Mo contents from 1 to 20 wt %. Based on the aforementioned results, the following conclusions can be drawn:

Ti–5Sn exhibits a hexagonal α′ phase. With a 1 to 5 wt % Mo addition to the Ti–5Sn alloy, the ternary alloys remained a single α′-Ti phase. As the Mo content was 7.5 wt %, the orthorhombic α″ phase was found. An increase of Mo content to 10 wt % or greater, the bcc β phase was entirely retained. Among the ternary Ti–5Sn–*x*Mo alloys, no peaks of the ω phase were found because Sn can effectively suppress the ω phase formation.

The ternary Ti–5Sn–*x*Mo alloys (360–423 HV) exhibited greater microhardness than the other metals tested in this study. Among them, Ti–5Sn–7.5Mo (365 HV) had a slightly lower hardness, while Ti–5Sn–20Mo (423 HV) had the greatest microhardness.

The bending strengths of all Ti–5Sn-based alloys (1643–2147 MPa) were significantly higher than that of c.p. Ti (844 MPa). The Ti–5Sn–7.5Mo alloy had a relatively low strength. Among β-phase Ti alloys, Ti–5Sn–20Mo exhibited the greatest strength, which was attributed to the decreased grain size and the solid solution effect of alloying elements.

The α″-phase Ti–5Sn–7.5Mo (80 GPa) had the lowest elastic modulus. In β-phase Ti alloys, the Ti–5Sn–10Mo (85 GPa) had the lowest elastic modulus, while the Ti–5Sn–20Mo (134 GPa) had the highest one.

The α″-phase Ti–5Sn–7.5Mo (27.4°) and β-phase Ti–5Sn–10Mo (37.8°) exhibited greater elastic recovery capability.

The optimal combined properties of microhardness, ductility, elastic modulus, and elastic recovery capability of both Ti–5Sn–7.5Mo and Ti–5Sn–10Mo alloys seem to be promising candidates for better implant materials.
